# Behavioral and biological divergence in monozygotic twin pairs discordant for autism phenotypes: A systematic review

**DOI:** 10.1111/jcv2.12017

**Published:** 2021-06-26

**Authors:** Lynnea Myers, Pei‐Yin Pan, Karl Lundin Remnélius, Janina Neufeld, Peter B. Marschik, Ulf Jonsson, Sven Bölte

**Affiliations:** ^1^ Department of Women's and Children's Health Karolinska Institutet Center of Neurodevelopmental Disorders Centre for Psychiatry Research Karolinska Institutet & Stockholm Health Care Services Stockholm Sweden; ^2^ Department of Nursing Gustavus Adolphus College St. Peter Minnesota USA; ^3^ Department of Child and Adolescent Psychiatry and Psychotherapy University Medical Center Göttingen & Leibniz Science Campus Göttingen Germany; ^4^ Department of Phoniatrics D –Interdisciplinary Developmental Neuroscience Medical University of Graz Graz Steiermark Austria; ^5^ Department of Neuroscience, Child and Adolescent Psychiatry Uppsala University Uppsala Sweden; ^6^ Department of Child and Adolescent Psychiatry Stockholm Health Care Services Stockholm Sweden; ^7^ Curtin Autism Research Group School of Occupational Therapy, Social Work and Speech Pathology Curtin University Perth Western Australia Australia

**Keywords:** autism spectrum disorders, discordant, etiology, monozygotic, non‐shared environment, review, twins

## Abstract

**Background:**

Non‐shared environment (NSE) effects account for around one‐third of the etiology of autism spectrum disorder (ASD). However, the knowledge of mechanisms and phenotypic profiles associated with NSE in ASD is scarce.

**Methods:**

A systematic search was conducted using Embase, MEDLINE, and PsycINFO for studies published in English between 1990 and August 2020 using co‐twin control design to compare behavioral and biological phenotypes among monozygotic (MZ) twin pairs concordant/discordant for ASD, clinical autism symptoms, or autistic traits. Risk of bias was assessed through a modified Newcastle–Ottawa Scale.

**Results:**

Twenty six articles were included. Differential DNA methylation and gene expression were found among ASD discordant twins; however, genetic results were inconsistent. Neurological disorders and early medical events were associated with ASD and autistic traits, while no within pair differences were found for minor physical anomalies or head circumference. Structural and functional brain imaging studies and research on social and other cognitive/behavioral functions were inconclusive. Risk of bias assessment found that all studies used the same exposure (or outcome) measures to collect data for participants and most used either secure health‐related records or structured interviews for ascertainment of exposure; however, only a handful of studies representative of the population from which they were drawn. Formal assessment of risk of publication bias (i.e., funnel plot) was not possible.

**Conclusions:**

Our results suggest that NSE in ASD could be associated with heterogeneous postzygotic genetic mechanisms and manifest as a range of biological and behavioral phenotypes. Extant findings were limited by relatively few studies, small sample sizes, and methodological diversity. More research is needed on co‐occurring biological and behavioral phenotypes using a consistent format for designing, analyzing, and reporting MZ ASD discordant twin studies in order to further examine the role of NSE in the etiology of ASD.

## Graphical abstract

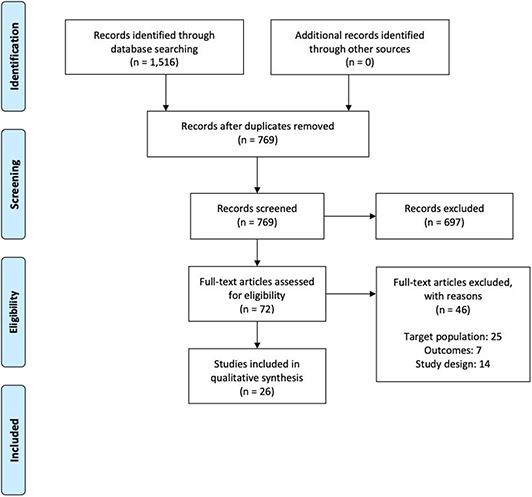

## INTRODUCTION

**Key points**

- Studies on behavioral and biological systems using co‐twin control design in monozygotic (MZ) twins discordant for autism spectrum disorder (ASD), clinical autism symptoms, or autistic traits might elucidate non‐shared environment (NSE) contributions to etiological pathways in autism.
- The co‐occurring phenotypes that were explored in the studies included in this review in relationship to ASD were genetic/biochemical, brain structural and functional, cognitive/behavioral, and physical.
- Only a few studies examined exclusively MZ ASD discordant twins.
- More studies using the MZ ASD discordant twin design with consistent standards for how the study is performed and reported are needed to explore the impact of NSE in autism, thereby providing insight into etiological mechanisms.

Autism spectrum disorder (ASD) is a complex, heterogeneous neurodevelopmental condition defined by alterations in social communication and interaction, as well as repetitive and restricted behaviors (American Psychiatric Association, 2013; Centers for Disease Control and Prevention, 2020). Despite substantial heritability for ASD (Tick et al., 2016), variance is attributable to environmental influence (Bölte et al., 2019), particularly non‐shared environment (NSE). NSE refers to “environmental influences that make children growing up in the same family different, and can include epigenetic process, gene expression, some de novo mutations, illnesses, intra‐ and extra‐uterine environment and measurement error” (Ronald & Hoekstra, 2011, p. 264). NSE may also include non‐definable environmental factors or stochasticity (Plomin, 2011). Estimates of NSE effects in ASD range between 16% and 33.6% (Bai et al., 2019; Lichtenstein et al., 2010; Sandin et al., 2017). Hypothesized NSE for ASD include pre‐, peri‐ and post‐natal factors (Lai et al., 2014; Lord et al., 2020), of which low birthweight, birth defects, perinatal hypoxia, and respiratory stress have shown to be associated with ASD beyond familial confounding (Carlsson et al., 2020). To investigate the contribution of NSE to within‐family differences, three essential elements for the research design are needed: (1) document differential experiences, which requires the construction of measures of the environment that are specific to each child in the family; (2) document the association between such differential experiences and differential outcomes; and (3) investigate the extent to which associations between differential experiences and differential outcomes are causal (Rowe & Plomin, 1981, as cited in Plomin et al., 2001). For environmental exposures, including NSE, it has been noted that genetic factors and critical time periods may affect whether or not exposures result in ASD (Schmidt et al., 2014).

The co‐twin control design with monozygotic (MZ) twins discordant for ASD and associated phenotypes may be a feasible strategy to examine the effects of NSE. The design implicitly controls for shared genetics and environment, and other unmeasured common confounders as well (Ronald & Hoekstra, 2011; Zwijnenburg et al., 2010). NSE may point to causes or consequences of a disease process that are independent of shared genetics or environment. This includes epigenetics, de novo genetic mutations or copy number variations, differences in brain morphology, and/or cognitive or behavioral differences that could be causal for heterogeneous/discordant outcomes (Ronald & Hoekstra, 2011; Zwijnenburg et al., 2010). For example, if a phenotype shows a within pair association with the diagnosis of ASD or the severity of autistic traits or symptoms in the affected MZ discordant twin, then the mechanisms underlying the phenotype are subsumed to be involved in the etiological pathways of autism. Based on this hypothesis, we defined phenotypes in this review as the biological and behavioral manifestations which can be measured in twins, including genetics, biochemical profiles, brain structure and function, cognitive/behavioral performance, and physical features and/or disorders.

For the last three decades, studies using twin modeling to investigate the etiology of ASD are increasing (Bailey et al., 1995; Bölte et al., 2014; Isaksson et al., 2018; Ronald & Hoekstra, 2011). Population‐based twin studies in large samples of MZ and dizygotic (DZ) twin pairs have provided estimates regarding the contribution of NSE to the variation of autistic traits and the liability to development of ASD. However, the specific profiles of NSE are still unclear. Therefore, this systematic review sought to identify studies of co‐occurring phenotypes in samples of MZ twins discordant for ASD diagnosis, clinical autism symptoms, and autistic traits. Because studies exclusively examining MZ ASD discordant twins alone are quite rare, studies with mixed samples of MZ discordant twins along with DZ discordant and/or MZ concordant twins were also included to increase the number of studies included in this review for power. The objective of this review is to provide direct or indirect evidence for behavioral and biological functions and systems possibly affected by NSE in autism; thereby, generating new or corroborating and specifying existing etiological hypotheses of the condition.

## METHOD

This review followed the Preferred Reporting Items for Systematic Reviews and Meta‐analyses (PRISMA; Moher et al., 2009). The protocol was registered in PROSPERO: International Prospective Register of Systematic Reviews (CRD42020206131, https://www.crd.york.ac.uk/prospero/display_record.php?RecordID=206131).

### Search strategy

A systematic search was conducted using MEDLINE (Ovid), Embase, and PsycINFO (Ovid) databases with the assistance of Karolinska Institutet Library for articles published in English between 1990 and August 12, 2020. A corresponding manual search was performed by scanning reference lists of included articles. Appendix S1 provides complete details regarding the search strategy by database.

### Study eligibility criteria

For inclusion, studies had to use co‐twin control design and examine biological or behavioral phenotypes in MZ twins discordant for ASD diagnosis, clinical autism symptoms, or autistic traits. Eligible studies defined categorical ASD diagnosis using DSM‐III, DSM‐IV(‐TR), DSM‐5, ICD‐9, or ICD‐10 or clinical autism symptoms or autistic traits using standardized scales providing quantifications of autism phenomenology (i.e., Autism Diagnostic Observation Schedule [ADOS‐2, Lord et al., 2012], Autism Diagnostic Interview‐Revised [ADI‐R, Rutter et al., 2003], Social Responsiveness Scale‐2 [SRS‐2; Constantino, 2005], Childhood Autism Spectrum Test [CAST, Scott et al., 2002]). We also included data derived from samples of autism discordant pairs regardless of zygosity and MZ pairs regardless of concordance of autism if the studies performed a conditional generalized estimating equation (GEE). The GEE model allows to control for all the shared confounders within each twin pair, including genetic components, age, sex, socioeconomic status, and early family experience, etc (Carlin, Gurrin, Sterne, Morley, & Dwyer, 2005). In this way, the specific association between the co‐occurring phenotypes and ASD among these twin pairs reflects the role of NSE and the possible common etiological pathways of the phenotypes in question.

Articles were excluded if they did not include any MZ discordant twins or were case studies where both twins were diagnosed with ASD or had similar levels of clinical autism symptoms or autistic traits. Studies that failed to describe and/or use validated methods for the diagnosis of ASD or autistic traits/symptoms were excluded, along with studies that failed to describe validated methods of zygosity determination. Studies solely comparing MZ discordant twins with either DZ discordant pairs and/or a control sample of typically developing twins and/or control individuals were excluded. Finally, studies without relevant outcome data for phenotypic features or focusing on heritability or concordance of diagnosis estimates alone were excluded.

### Study selection, data extraction, and risk of bias assessment

After removal of duplicates, all titles and abstracts of remaining articles were independently screened by the first and second author (LM and PP) for eligibility. Articles passing stage one screening were then assessed in full‐text for eligibility (by LM and PP). Consensus on data extraction was achieved between the two raters with any disagreements resolved by the senior author (SB). Primary data extraction from the articles was performed by the first author (LM). A secondary check of the data extraction was completed by one of three co‐authors (PP, KLR and JN). Consensus on data extraction was achieved between the two raters with any disagreements resolved by the senior author. See Table S1 for excluded articles and Table S2 for data extraction elements.

### Quality assessment

The first author rated individual study quality using the Newcastle‐Ottawa Quality Assessment Scale (NOS; Wells et al., 2019). The case‐control version of the NOS was used, but modified for twin studies through the removal of the ratings for selection and definition of controls and comparability, resulting in a five‐item scale. Studies with data for more than one exposure/outcome resulted in exposure/outcome being rated separately. A secondary check of the quality assessment was completed by one additional co‐author (PP, KLR and JN).

### Synthesis

A narrative synthesis of the results of included studies was performed. The phenotypes were grouped into four categories according to their nature: genetics/biochemical, brain structural and functional, cognitive/behavioral, and physical. For each category, the results of within pair associations and comparisons within discordant pairs were presented separately. No meta‐analyses were performed as data from at least two separate cohorts of comparable design were not available.

## RESULTS

**FIGURE 1:**
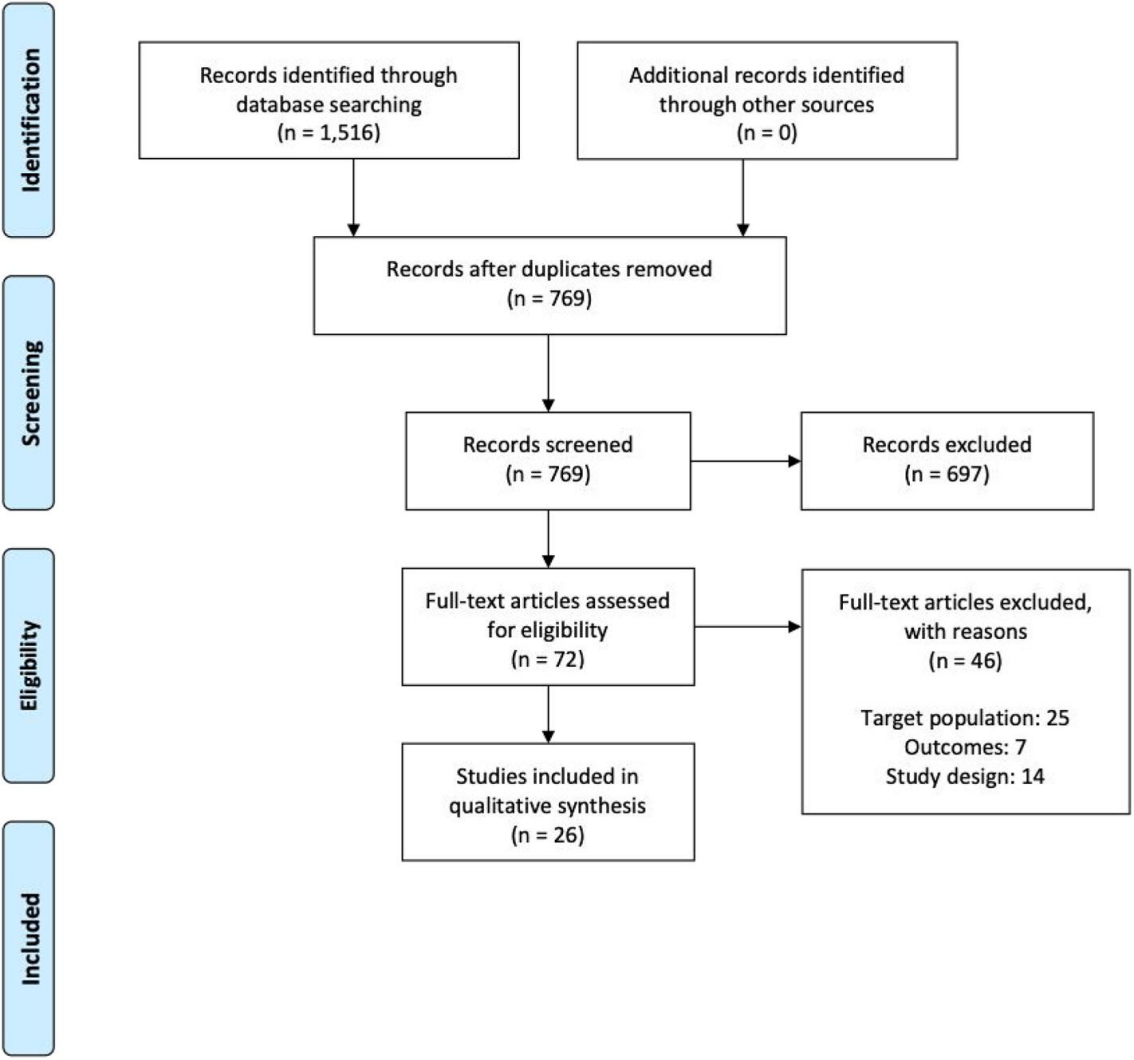
PRISMA flow diagram

Twenty‐six studies were included in this review (Figure 1). Figure S1 illustrates NOS ratings for the included articles. Additional study details related to demographics and study design and methods can be found in Tables S3 and S4, respectively.

### Co‐occurring genetic/biochemical phenotypes

Eight studies, including four case series/reports, investigated the intra‐pair differences of genetic phenotypes (Table 1): four focused on DNA variations such as copy number variations (CNVs), single nucleotide polymorphisms (SNPs), or insertions/deletions (indels) (Huang et al., 2019; Laplana et al., 2014; Morimoto et al., 2017; Stamouli et al., 2018), two focused on DNA methylation (Liang et al., 2019; Wong et al., 2014), and two focused on gene expression (Hu et al., 2006; Saffari et al., 2019).

**TABLE 1:** Studies with co‐occurring genetic/biochemical phenotypes

	*Studies with samples of MZ discordant pairs but not using within pair analysis (conditional GEE)*					
*Discordant type*	*Coexisting phenotypes*	*Study*	*Tissue source*	*N of pairs (% male)*	*Ages, mean* ± *SD*	*Main results (difference between affected/more severe twins and the co‐twins)*
ASD	Patterns/levels of manganese/zinc/lead	Arora et al. (2017)	Teeth	3 (NR)	14.7 ± 3.9 (NR)^a^	Different patterns/levels of fetal and postnatal manganese/zinc/lead recorded in teeth
	DNA methylation	Liang et al. (2019)	Blood	5 (60.0)	8.2 ± 3.7 (2–12)	2397 differentially methylated genes identified, enriched in neurotrophin signaling pathway
	Gene expression (transcription differences)	Saffari et al. (2019)	Blood	5 (54.5)^b^	12 ± NR (NR)	3 genes showed differential expression, with functions of antigen binding and immunoglobulin receptor binding, transmembrane signaling, and methylation
	Rare CNVs	Stamouli et al. (2018)	Saliva	13 (61.5)	14.0 ± NR (9–20)	No non‐shared postzygotic de novo CNVs found
Autistic trait/symptoms	DNA methylation	Wong et al. (2014)	Blood	S: 9 (33.3); R: 10 (70.0); C: 9 (66.7)^c^	8 and 15^d^ ± NR (NR)	Significant within pair DNA methylation differences: 9 for S, 9 for R, and 8 for C; enriched in genes previously implicated in etiology of ASD
	***Case report/series with MZ discordant pairs***					
*Discordant type*	*Coexisting phenotypes*	*Study*		*N of pairs (% male)*	*Ages*	*Main results*
ASD	Gene expression	Hu et al. (2006)	Blood	3 (100.0)	6, 8, and 16 years	44 genes differentially expressed between the more severely affected twins and cotwins, enriched in genes involved in neurological development, function, or disease
	DNA variation (SNPs, indels, and CNVs)	Huang et al. (2019)	Blood	3 (NR)	NR	Discordant variation observed in 2 twin pairs, referring to a total of 2174 genes enriched in neurodevelopmental processes
		Morimoto et al. (2017)	Blood	1 (100.0)	11	No significant findings of somatic cell mosaic mutations
	Germinal and somatic CNV regions	Laplana et al. (2014)	Blood, saliva, hair follicle	1 (100.0)	24	No significant differences in CNV regions between twins explain the observed differences on clinical profiles

Abbreviations: ASD, autism spectrum disorder; CNV, copy number variant; GEE, generalized estimating equation; MZ, monozygotic; NR, no report; RRBIs, restricted and repetitive behaviors and interests; SNP, Single nucleotide polymorphisms.

^a^ Data of the whole sample (20 individuals, including 3 MZ pairs and 4 DZ pairs).

^b^ Five pairs and one individual (unaffected twin).

^c^ Social autistic traits (S): N = 9 MZ pairs; autistic RRBIs (R): N = 10 MZ pairs; and communication autistic traits (C): N = 9 MZ pairs.

^d^ Dimensional ASD traits were assessed at age 8 years, whole blood sample at 15 years.

Differential DNA methylation was found among twins discordant for ASD diagnosis and autistic traits. In relationship to ASD diagnosis, genes identified with differential methylation were mainly involved in the neurotrophin signaling pathway (Liang et al., 2019), while the genes linked to trait‐discordance were ASD‐associated genes (e.g., NLGN2, SNRPN, SNURF, etc.; Wong et al., 2014). Two studies focusing on gene expression both reported differential expression between affected twins and their co‐twins (Hu et al., 2006; Saffari et al., 2019); however, the functions of these identified genes varied across studies. Among the four studies investigating DNA variations (Huang et al., 2019; Laplana et al., 2014; Morimoto et al., 2017; Stamouli et al., 2018), only Huang et al. (2019) reported discordant variation found in two of three pairs in their sample, while the remaining three studies found no significant within pair differences for de novo DNA variations.

In terms of the biochemical phenotypes, Arora et al. (2017) reported differing patterns/levels of fetal and postnatal manganese/zinc/lead uptake in primary teeth between three pairs of ASD affected twins and their co‐twins.

### Co‐occurring brain structural and functional phenotypes

A total of five studies focused on co‐occurring structural and functional brain phenotypes (Table 2). These two group studies (Cauvet et al., 2019; Neufeld et al., 2018) investigated within pair relationships between ASD and brain imaging measures with largely overlapping samples of MZ concordant and discordant twin pairs. Neufeld et al. (2018) revealed increased intrinsic connectivity between hubs of the salience network in the more autistic twins compared with their co‐twins, while Cauvet et al. (2019) identified reduced regional volume and surface area specifically in more autistic female twins compared with their co‐twins. The three included case studies yielded mixed results. In Kates et al. (1998), the affected male twin showed reduced global and regional brain volume compared with his co‐twin. Belmonte and Caper (2006) did not find such differences in a male twin pair discordant for clinical autism symptom severity, but concordant for diagnosis; however, they did find more condition‐specific brain activation during visual task performance in the higher functioning twin. Finally, Conti et al. (2015) found primarily reduced connectivity in a male twin diagnosed with ASD compared to his cotwin without diagnosis, especially affecting left frontoparietal and frontotemporal connections.

**TABLE 2:** Studies with co‐occurring brain structural and functional phenotypes

*Studies using within pair analysis (conditional GEE)*					
*Discordant type*	*Exposures (E)/outcomes (O)*	*Study*	*N of pairs (% male)*	*Ages, mean* ± *SD*	*Main results*
MZ concordant/discordant pairs					
Autistic trait/symptoms	Regional cortical volume, surface area and thickness (O)	Cauvet et al. (2019)	49 (55.1)^a^	Female: 16.2 ± 3.4 (9–23.7)^b^	In females only:
				Male: 15.5 ± 2.8 (10.7–23)^b^	Volume^c^: β‐range = −29.8 to −7.1, p‐range = 0.007 to 0.002
					Surface area^c^: β‐range = −2.5 to – 1.5, p‐range: 0.03 to 0.00002
					Thickness^d^ of the right lateral superior temporal gyrus: β = 2.9, p = 0.008
	Resting state connectivity between regions of interest (O)	Neufeld et al. (2018)	46 (60.9%)^a^	16.95 ± 3.09 (NR)^a^	Salience network: β = 0.0045, p = 0.005^e^
					Default mode network: β = − 0.0004, p = 0.842^f^
***Case report/series with MZ discordant pairs***					
*Discordant type*	*Coexisting phenotypes*	*Study*	*N of pairs (% male)*	*Ages, mean* ± *SD*	*Main results*
Autistic trait/symptoms	Brain activation during visual task and regional brain volumes	Belmonte et al. (2006)	1 (100.0)	13.45	fMRI: More condition‐specific brain activation in the higher functioning twin, but no clear difference between twins in brain volume
	Structural brain connectivity	Conti et al. (2015)	1 (100.0)	5.2 (NA)	Of 1122 fiber tracts, fractional anisotropy >10% different in 136 in twin with ASD: Lower in 125 (left hemisphere, especially frontoparietal and frontotemporal) and higher in 11
	Total and regional brain volumes measured manually	Kates et al. (1998)	1 (100.0)	7.5 (NA)	Total cerebral volume was 14.7% smaller in twin with ASD and smaller regional volumes (caudate, amygdala, hippocampal, and cerebellar vermis)

Abbreviations: ASD, autism spectrum disorder; GEE, generalized estimating equation; MZ, monozygotic; NA, not applicable; NR, no report.

^a^ All MZ twins.

^b^ In the whole investigated sample.

^c^ Adjusted for IQ, handedness, and total brain volume.

^d^ Adjusted for IQ and handedness.

^e^ Adjusted for IQ and head motion.

^f^ Adjusted for IQ.

### Co‐occurring cognitive/behavioral phenotypes

Five studies (Cauvet et al., 2020; Humphrey et al., 2004; Isaksson, Taylor, et al., 2019; Isaksson, Van't Westeinde, et al., 2019; Neufeld et al., 2020) reported cognitive/behavioral phenotypes in relation to ASD diagnosis, clinical autism symptoms, or autistic traits (Table 3), four of which used largely overlapping samples from the Roots of Autism and attention deficit‐hyperactivity disorder Twin Study in Sweden (RATSS; Bölte et al., 2014). Three of the studies investigated social cognition: one found a negative association between social cognition and ASD within MZ and DZ discordant ASD pairs (Cauvet et al., 2020), while two studies conducting within pair analyses in MZ pairs found no significant association between social cognition and ASD diagnosis (Isaksson, Taylor, et al., 2019; Isaksson, Van't Westeinde, et al., 2019). However, Isaksson, Van't Westeinde, et al. (2019) reported that social cognition was negatively associated with both autistic traits and autism symptoms within MZ pairs. Neufeld et al. (2020) assessed global visual processing with the Fragmented Pictures Test and found an association between ASD diagnosis (but not autistic traits) and a need for more visual information during global visual processing. In the case study by Humphrey et al. (2004) following a male MZ pair discordant for both ASD and autism symptoms from 18 to 36 months of age, the twin fulfilling criteria for ASD displayed mild to moderate intellectual disability, whereas his co‐twin had slightly higher intelligence.

**TABLE 3:** Studies with co‐occurring cognitive/behavioral phenotypes

*Studies using within pair analysis (conditional GEE)*					
*Discordant type*	*Exposures (E)/Outcomes (O)*	*Study*	*N of pairs (% male)*	*Ages, mean* ± *SD*	*Main results*
MZ/DZ discordant pairs					
ASD	Social cognition ability (O) (movie for the assessment of social cognition)	Cauvet et al. (2020)	18^a^ (55.5)	19.6 ± NR (12.5–31)^b^	β = −3.590, 95% CI 6.778, −0.408, p = 0.027^c^
MZ concordant/discordant pairs					
ASD	Social cognition ability (O) (reading the mind in the eyes test)	Isaksson et al. (2019a)	97 (52.6)^d^	17.07 ± 5.53 (8–29)^d^	β = −1.22 (−7.33 to 4.89), p = 0.70^e^
	Social cognition ability (E) (movie for the assessment of social cognition)	Isaksson et al. (2019b)	61^f^ (42.6)^d^	19.4 ± 4.8 (12–31)^b^	β = −0.28 (−0.56 to 0.01), p = 0.058^c^
	Global visual processing (picture completion in the fragmented pictures test) (O)	Neufeld et al. (2020)	26^g^ (60.0)	17.3 ± 5.7 (9–29)^d^	β = 2.49, p = 0.04^c^
Autistic trait/symptoms	Social cognition ability (O) (reading the mind in the eyes test)	Isaksson et al. (2019a)	97 (52.6)^d^	17.07 ± 5.53 (8–29)^d^	β = −0.01 (−0.12 to 0.10), p = 0.88^e^
	Social cognition ability (E) (movie for the assessment of social cognition)	Isaksson et al. (2019b)	61^f^ (42.6)^d^	19.4 ± 4.8 (12–31)^b^	Traits: β = −2.08 (−2.95 to −1.20), p < 0.001^c^ ^,^ ^i^ ^(^ ^g^ ^)^
					Symptoms: β = −0.11 (−0.21 to −0.18), p = 0.021^c^
	Global visual processing (picture completion in the fragmented pictures test) (O)	Neufeld et al. (2020)	87^g^ ^(^ ^h^ ^)^ (52.4)^b^	17.3 ± 5.7 (9–29)^d^	NS: β = 0.03, p = 0.21^c^
***Case report/series with MZ discordant pairs***					
*Discordant type*	*Coexisting phenotypes*	*Study*	*N of pairs (% male)*	*Ages, mean* ± *SD*	*Main results*
ASD	Cognitive function (mullen scales of early learning)	Humphrey et al. (2004)	1 (100)	Followed between 18 and 36 months age	Twin with ASD had developmental quotient in mild‐moderate range of intellectual disability (ID), while co‐twin (not diagnosed with ASD) had low average to borderline ID range^i^

Abbreviations: ADHD, attention deficit‐hyperactivity disorder; ASD, autism spectrum disorder; DZ = dizygotic; GEE, generalized estimating equation; MZ, monozygotic; NR = no report; NS = not significant.

^a^ 7 MZ and 11 DZ pairs.

^b^ In the whole investigated sample.

^c^ Adjusted for IQ.

^d^ All MZ twins.

^e^ Adjusted for IQ, ADHD, ID, and anxiety/depression disorders.

^f^ 18 discordant pairs.

^g^ MASC subscales hypermentalizing (β = 1.97 (0.41 to 3.52), *p* = 0.013), hypomentalizing (β = 2.75 (0.25 to 5.24), *p* = 0.031), and concrete mentalizing (β = 2.39 (0.34 to 4.45), *p* = 0.023) were also associated with autistic traits. New i.

^h^ 16 discordant pairs.

^i^ Twin with ASD also received higher scores on ADOS (at ages 18, 24 and 36 months) and ADI‐R (at age 36 months) than co‐twin without ASD diagnosis.

### Co‐occurring physical phenotypes

A total of eight studies focused on co‐occurring physical phenotypes (Table 4), including two examining physical problems, three examining early medical events, and three examining physical anomalies/characteristics. Four studies performed conditional GEEs to examine within pair associations (Myers et al., 2017, 2018; Pan et al., 2020; Willfors et al., 2017); however, two of them did not define trait discordancy in their analyses (Myers et al., 2017, 2018). For the within pair effects, neurological disorders were found to be associated with both ASD and autistic traits (Pan et al., 2020) and early medical events, defined in Willfors et al. (2017), were associated with autistic traits. Neither the number of minor physical anomalies nor the ratio of the second finger digit with the fourth finger digit (2D:4D ratio) had significant associations with categorical or dimensional ASD (Myers et al., 2017, 2018). Four studies, including one case report, compared differences in physical phenotypes between affected twins and co‐twins within ASD discordant pairs. Affected twins with ASD, in comparison to unaffected co‐twins, were found to have more perinatal morbidity (Bailey et al., 1995; Froehlich‐Santino et al., 2014), more early medical events (Willfors et al., 2017), and more persistent seizures (Humphrey et al., 2004). There was no within pair difference of head circumference (Froehlich et al., 2013).

**TABLE 4:** Studies with co‐occurring physical phenotypes

*Studies using within pair analysis (conditional GEE)*					
*Discordant type*	*Exposures (E)/Outcomes (O)*	*Study*	*N of pairs (% male)*	*Ages, mean* ± *SD*	*Main results*
MZ discordant pairs					
ASD	Neurological problems (E)	Pan et al. (2020)	18 (55.6)	15.3 ± 4.9 (9–28)	OR = 3.15 (1.20–8.30), p = 0.020
Autistic trait/symptoms	Neurological problems (E)	Pan et al. (2020)	64 (48.4)	16.7 ± 5.5 (8–29)	β = 10.44, p = 0.006^a^
	Early medical events (E)	Willfors et al. (2017)	54 (55.5)	14.9 ± NR (8–28)	Cumulative load of early medical events: β = 78.18^b^, p = 0.002
					Cumulative load of early medical events, excluding dysregulation variable^c^: β = 54.42^b^, p = 0.005
					Dysregulation: β = 31.75^b^, p = 0.03
					Birthweight: β = −0.01^b^, p = 0.05
MZ concordant/discordant pairs					
ASD	Number of minor physical anomalies (MPAs) (E)	Myers et al. (2017)	18 (62.5%)	14.1 ± 3.4 (9–23)^d^	OR 1.43 (0.47–6.45), p = 0.413^e^
					OR 2.71 (0.83–8.81), p = 0.097^f^
					OR 2.71 (0.83–8.81), p = 0.097^g^
	Overall hand 2D:4D ratio (O)	Myers et al. (2018)	19 (62.2%)	16.2 ± 5.2 (8–29)^d^	β = −0.010 (−0.024 to 0.003)^f^
					β = −0.010 (−0.023 to 0.003)^g^
Autistic trait/symptoms	Number of minor physical anomalies (MPAs) (E)	Myers et al. (2017)	51 (56.9)^d^	14.1 ± 3.4 (9–23)^d^	β = 1.88 (−1.41 to 5.17), p = 0.263^e^
	Overall hand 2D:4D ratio (O)	Myers et al. (2018)	70 (55.4)^d^	16.2 ± 5.2 (8–29)^d^	β = 0.0001 (−0.0002 to 0.0003)
***Studies with samples of MZ discordant pairs but not using within pair analysis (conditional GEE)***					
*Discordant type*	*Coexisting phenotypes*	*Study*	*N of pairs (% male)*	*Ages, mean* ± *SD*	*Main results (difference between affected/more severe twins and the co‐twins)*
ASD	Early medical events	Bailey et al. (1995)	10 (77.8)^d^	NR	5 pairs with biological hazards and differences only in affected twins^i^
		Froehlich‐Santino et al. (2014)	20 (75.0)	NR (4–18)^d^	3 pairs discordant for respiratory distress (only in affected twins)
		Willfors et al. (2017)	13 (61.5)	14.4 ± NR (9–20)	Dysregulation: Z = −2.56, p = 0.011
					Birth weight: Z = −2.20, p = 0.028
					Cumulative load of early medical events: Z = −2.85, p = 0.004
	Head circumference (HC)	Froehlich et al. (2013)	21 (76.2)	9.7 ± 3.4 (4–18)^d^	NS difference in HC between affected twins and co‐twins; 2 pairs discordant for macrocephaly
***Case report/series with MZ discordant pairs***					
*Discordant type*	*Coexisting phenotypes*	*Study*	*N of pairs (% male)*	*Ages, mean* ± *SD*	*Main results*
ASD	Number/location of tubers, epilepsy	Humphrey et al. (2004)	1 (100)	Followed between 18 and 36 months age	Twin with ASD judged to have more extensive brain involvement despite fewer tubers than co‐twin and earlier onset and more persistent epileptic seizures

Abbreviations: ADHD, attention deficit‐hyperactivity disorder; ASD, autism spectrum disorder; GEE, generalized estimating equation; MZ, monozygotic; NDDs, neurodevelopmental disorders; NR, no report; NS, not significant; OR = odds ratio; RDS, respiratory distress syndrome.

^a^ Adjusted for IQ, ADHD, and other NDDs.

^b^ One‐tailed test.

^c^ Dysregulation in the first year of life was defined as feeding and sleeping problems, excessive crying, and worrying; new b.

^d^ In the whole investigated sample.

^e^ Adjusted for IQ.

^f^ Adjusted for ADHD.

^g^ Adjusted for other NDDs.

^h^ Data of the whole MZ sample (27 pairs).

^i^ Multiple congenital anomalies, neonatal convulsions, RDS and cardiac arrest, delay second birth, and lighter birthweight.

### Risk of bias within and across studies

The risk of bias is plotted in Figure S1 using the modified NOS for each study. Of note, all studies reported using the same exposure (or outcome) measures to collect data for participants within the studies and most used either secure health‐related records or structured interviews for ascertainment of exposure. Only five studies were determined to have cases representative of the population from which they were drawn. Formal assessment of risk of publication bias (i.e., funnel plot) was attempted for included studies, but due to the heterogeneity of the study outcomes, the limited number of studies providing appropriate information for calculating effect sizes, and/or some biological or behavioral phenotypes being used as exposures in statistical calculations, we were not able to generate meaningful plots.

## DISCUSSION

The results of this review suggest that contributions of NSE in ASD could involve heterogeneous postzygotic genetic mechanisms, and the associated phenotypes include brain structure and function, cognitive/behavioral profiles, physical comorbidities, and early medical adversities. However, there were limited studies identified for this review and the small sample size affected statistical power of the review. Methodological diversity, including differences in the definition of discordance for ASD across the studies, also means the findings in this review should be interpreted with necessary caution. There is a definite need for further investigations using larger, well‐designed and reported studies on MZ twins discordant for ASD, clinical autism symptoms, or autistic traits in samples of high generalizability in order to corroborate, reject, or refine results of this review. In order to guide future research, we summarize findings by co‐occurring phenotypes explored and suggest strategies for how future studies should be performed and reported.

### Co‐occurring phenotypes

#### Genetic/biochemical

For co‐occurring genetic and biochemical phenotypes, only studies with small sample sizes were found. Most of them reported differences in genetics between the affected twins (or twins with more severe symptoms) and their cotwins. However, to what extent these identified differences contributed to the etiology of ASD, as well as the generalizability of these results were unclear. Moreover, the results were inconsistent across these studies, reflecting the heterogeneity of participants, methodology, and etiology of ASD. Blood was the primary tissue source for genetic testing in the studies reviewed. A recent study examined the correlation between genome‐wide methylation findings found in brain tissue compared with blood, saliva, and buccal samples and found strong correlations for methylation findings from blood and saliva samples with brain tissue samples (Braun et al., 2019); thereby, providing some support for the use of these peripheral tissue samples in genetic analyses.

#### Structural and functional brain imaging

Only a handful of neuroimaging studies were included in this review. These studies focused on a range of measures (i.e., brain volume, surface area, structural or intrinsic brain connectivity, or task‐related brain activation). One group study (Cauvet et al., 2019) and one case study (Kates et al., 1998) found reduced regional cortical volume in association with ASD. However, the group study found a significant association only in females and the case study assessed a single male twin pair.

#### Cognitive/behavioral

Of the studies assessing cognitive/behavioral phenotypes, social cognition was the phenotype studied most frequently (Cauvet et al., 2020; Isaksson, Taylor, et al., 2019; Isaksson, Van't Westeinde, et al., 2019) and only Cauvet et al. (2020) found an association between social cognition and ASD. The studies in this area had methodological differences related to both samples researched and measures used for social cognition, tapping different domains and levels of complexity in social cognition with potentially differing genetic/environmental underpinnings.

#### Physical

For studies examining co‐occurring physical phenotypes, only a few studies used a conditional GEE. These studies mostly assessed largely overlapping samples (from RATSS). Several studies exploring physical phenotypes were case studies; therefore, the ability to conduct statistical analysis was limited.

### Design, analysis, and reporting for MZ ASD discordant twin studies

Further research using well‐designed studies of MZ discordant twin pairs for ASD, clinical autism symptoms, or traits of autism is needed to explore altered behavioral and biological systems influenced by NSE in the etiology of ASD. A major issue in this review was the varying definitions for ASD discordance used in the studies, resulting in the need to look specifically into each individual study for how discordance was defined. Some studies focused on qualitative differences based on categorical diagnoses, while others used quantitative differences on measures of ASD symptoms or traits to define discordance. No current standard exists for defining discordance/concordance in twins with ASD. In our review, studies stemming from RATSS most thoroughly described how discordance was determined in the studies, using both qualitative and quantitative differences to characterize twin pairs as concordant or discordant. In the literature, Gatz et al. (2000) discussed the challenges in defining discordance, particularly in twins with late‐onset disorders like Alzheimer’s disease. The authors specifically described the lack of a standard definition and how this can affect ways in which twin pairs are categorized and can be misclassified, which can statistically affect the ability to find significant differences in outcomes. In order to achieve better standardization and scientific quality, we propose the following guidelines for the design, analysis, and reporting of future MZ ASD discordant twin studies.

#### Design

To avoid risk of bias, samples should be more representative of all twin cases in a population, rather than applying selective recruitment or convenience sampling. For example, 80% of RATSS participants in Sweden are also part of national population‐based twin registries (Arora et al., 2017). Representativeness also includes samples that are more balanced in terms of gender and age ranges. Additionally, the recruitment of samples should be fully and transparently described and any bias or lack of representativeness in recruitment should be explicitly stated. Authors should thoroughly describe ways in which discordance/concordance was determined for twin pairs, including whether or not it was based on qualitative and/or quantitative measures (with specific cut‐offs, critical differences, or standard deviations used for quantitative measures). Additionally, when reporting results for social cognition, qualitative discordance should be reported for social‐communicative and non‐social/restricted and repetitive behaviors and interests dimensions separately. When possible, data related to chorionicity, if the twin pair shared a single or separate chorion, should also be collected and examined in relation to outcomes (Marceau et al., 2016). When providing demographic characteristics related to the sample, authors should include descriptions for MZ ASD discordant twins specifically, including the number of discordant twins, mean or median age, age range, age at diagnosis of ASD, gender ratio, race/ethnicity, and socioeconomic status. The methods used to diagnose ASD in participants should be based on gold standard or validated assessment instruments. Researchers should consider utilizing both ASD diagnosis and traits/symptoms as outcomes as a method to increase power and prevent some of the challenges related to relying on diagnostic or traits/symptoms information alone from a single source (i.e., parent report vs. clinician rating). Finally, to aid in the ability to assess risk of bias in publication using methods such as funnel plots, researchers should consistently report appropriate information (e.g., means and standard deviations) for both affected and unaffected groups related to outcomes of interest.

#### Analysis

For MZ discordant twin designs, analyses focusing on between and within pair associations should be prioritized over other statistical methods which do not account for the paired nature of data from twins. Additionally, analyses on purely MZ discordant samples should be provided in order to examine the effects of NSE. This would be in addition reporting results on DZ discordant and/or MZ concordant twins. These “mixed” samples include additional participants who are not as informative as MZ discordant twins alone and may lower the power to find an association. Authors should describe adjustments made to analyses for IQ, co‐occurring neurodevelopmental disorders, and other possible confounders. Finally, a description of how missing data was handled should be included.

#### Reporting

In the methods section, authors should describe how zygosity was determined for pairs and describe any with missing zygosity status. Authors should also describe the validity and reliability of measures used to collect data in the study and whether or not blinding was used. Finally, authors should clearly describe any drop‐outs of participants or those refusing to complete certain measures.

The methods used to diagnose ASD should be clearly reported, including the instruments and diagnostic criteria applied (as also recommended in Lord et al., 2020). Discordance should be specified as being based on ASD diagnosis and/or symptoms/traits. For dimensional definitions, authors should provide used cut‐offs, critical differences, standard errors of measurement, or standard deviations for discordance.

### Review strengths and limitations

This review represents, to the authors' best knowledge, the first to systematically review studies on MZ ASD discordant twins in order to identify potential NSE effects. Although the original intent of the review was to focus on studies examining co‐occurring phenotypes in MZ ASD discordant twins only, due to the limited number of studies using this design, the review expanded to include mixed samples of MZ discordant twins along with DZ discordant and/or MZ concordant twins to increase power. We acknowledge this adds “noise” to the results, but also allowed us to report findings on a larger number of studies in a field of ASD research where the availability of studies including exclusively MZ discordant twins is limited. Finally, it is important to note that findings from twin studies should generally be replicated in non‐twin samples using the case‐control design to allow for generalization of findings to other populations aside from twins.

## CONCLUSION

The current state of the research focusing on MZ ASD discordant twins is limited in its ability to provide insight into behavioral and biological system alterations driven by NSE contributions in the etiology of ASD; however, it does provide some guidance regarding factors that may be worth exploring, including genetic/biochemical, brain structural and functional, cognitive/behavioral, and physical. More MZ ASD discordant twin studies are needed using consistent design, analysis, and reporting, along with within pair analyses instead of group comparisons, to help advance the science into NSE.

## CONFLICT OF INTERESTS

All authors declare no direct conflict of interest related to this review; however Lynnea Myers, Pei‐Yin Pan, Karl Lundin Remnélius, Janina Neufeld, and Sven Bölte are authors of some of the included studies. Sven Bölte discloses that he has in the last three years acted as an author, consultant, or lecturer for Medice and Roche. He receives royalties for textbooks and diagnostic tools from Hogrefe, Kohlhammer, and UTB.

## ETHICAL STATEMENT

Since this systematic review did not directly involve human subjects, no ethical permission was needed.

## AUTHOR CONTRIBUTIONS

Sven Bölte and Ulf Jonsson conceptualized the study and Sven Bölte secured funding to support the study. Lynnea Myers, Pei‐Yin Pan, Karl Lundin Remnélius, Janina Neufeld, with the direction of Ulf Jonsson and Sven Bölte, performed the systematic review and analysis of findings. Lynnea Myers drafted the manuscript, with input and critical review from Pei‐Yin Pan, Karl Lundin Remnélius, Janina Neufeld, Peter B. Marschik, Ulf Jonsson, and Sven Bölte All authors read and approved the manuscript for submission

## Supporting information

Supporting information 1

## Data Availability

The data that support the findings of this study are available from the corresponding author upon reasonable request.
