# Supplementary material for: Behavioral and biological divergence in monozygotic twin pairs discordant for autism phenotypes: A systematic review
Source: JCPP Adv. 2021 Jun 26;1(2):e12017. doi: 10.1111/jcv2.12017 (PMC10242874; doi:10.1111/jcv2.12017)
Supplement: Supplementary file 1 — Supporting information 1 [file JCV2-1-e12017-s001.docx]

Online Supporting Information for: *Behavioral and Biological Divergence in Monozygotic Twin Pairs Discordant for Autism Phenotypes: A Systematic Review* – by Myers et al.

**Appendix S1.** Search Details.

Common search terms for MEDLINE, Embase, and PsycINFO: child development disorders, pervasive, Asperger*, autist*, autism*, autism spectrum disorder, ASD, PPD-NOS, pervasive child development disorder*, twin*, twins, twin studies, and diseases in twins.

1. MEDLINE

| Interface: Ovid  Date of Search: August 12, 2020  Number of hits: 557  Comment: In Ovid, two or more words are automatically searched as phrases; i.e. no quotation marks are needed | Field labels   - exp/ = exploded MeSH term - / = non exploded MeSH term - .ti,ab,kf. = title, abstract and author keywords - adjx = adjacent within x words, regardless of order - * = truncation of word for alternate endings |
| --- | --- |
| 1. exp Child Development Disorders, Pervasive/  2. (Asperger* or autist* or autism* or ASD or PDD-NOS or pervasive child development disorder*).ti,ab,kf. 3. or/1-2  4. exp Twins/  5. Diseases in Twins/  6. Twin Studies as Topic/  7. twin*.ti,ab,kf.  8. Twin Study.pt.  9. or/4-8  10. 3 and 9  11. 10 not (animals not humans).sh.  12. limit 11 to (english language and yr="1990 -Current")  13. limit 12 to (comment or congress or consensus development conference or consensus development conference, nih or editorial or interview or letter)  14. 12 not 13 | |

2. Embase

| Interface: embase.com  Date of Search: August 12, 2020  Number of hits: 633  Comment: Emtree is the controlled vocabulary in Embase | Field labels   - /exp = exploded Emtree term - /de = non exploded Emtree term - ti,ab = title and abstract - NEAR/x = adjacent within x words, regardless of order - * = truncation of word for alternate endings |
| --- | --- |
| ('autism'/exp OR asperger*:ti,ab,kw OR autist*:ti,ab,kw OR autism*:ti,ab,kw OR asd:ti,ab,kw OR 'pdd-nos':ti,ab,kw OR 'pervasive child development disorder*':ti,ab,kw)  AND  ('twins'/exp OR 'twin study'/de OR twin*:ti,ab,kw)  NOT  ([animals]/lim NOT [humans]/lim)  AND  [english]/lim AND [1990-2020]/py AND ([article]/lim OR [article in press]/lim OR [erratum]/lim OR [review]/lim OR [short survey]/lim) | |

3. PsycINFO

| Interface: Ovid  Date of Search: August 12, 2020  Number of hits: 326  Comment: In Ovid, two or more words are automatically searched as phrases; i.e. no quotation marks are needed | Field labels   - exp/ = exploded controlled term - / = non exploded controlled term - .ti,ab,id. = title, abstract and author keywords - adjx = adjacent within x words, regardless of order - * = truncation of word for alternate endings |
| --- | --- |
| 1. exp autism spectrum disorders/  2. (Asperger* or autist* or autism* or ASD or PDD-NOS or pervasive child development disorder*).ti,ab,id. 3. or/1-2  4. exp twins/  5. twin*.ti,ab,id.  6. or/4-5  7. 3 and 6  8. 7 not (animals not humans).sh.  9. limit 8 to (english language and yr="1990 -Current")  10. limit 9 to (chapter or dissertation or editorial or interview or letter or review-book)  11. 9 not 10 | |

**Table S1.** Studies from full-text review that were not included in the final sample and reasons why. A total of 46 articles were excluded from the final sample.

| **Reference** | **Reason for Exclusion** |
| --- | --- |
| Austin, C., Curtin, P., Curtin, A., Gennings, C., Arora, M., Tammimies, K., … & Bölte, S. (2019). Dynamical properties of elemental metabolism distinguish attention deficit hyperactivity disorder from autism spectrum disorder. *Translational Psychiatry*, *9*, 238. | Target population- No focus on MZ discordant ASD pairs |
| Bohm, H.V., Stewart, M.G., & Healy, A.M. (2013). On the Autistic Spectrum Disorder concordance rates of twins and non-twin siblings. *Medical Hypotheses*, *81*, 789–791. | Outcomes- No usable outcome measurement |
| Bölte, S., Willfors, C., Berggren, S., Norberg, J., Poltrago, L., Mevel, K., … & Lichtenstein, P. (2014). The Roots of Autism and ADHD Twin Study in Sweden (RATSS). *Twin Research and Human Genetics*, *17*, 164–176. | Outcomes- No usable outcome measurement |
| Brereton, E., Fassi, E., Araujo, G.C., Dodd, J., Telegrafi, A., Pathak, S.J., & Shinawi, M. (2018). Mutations in the PH Domain of DNM1 are associated with a nonepileptic phenotype characterized by developmental delay and neurobehavioral abnormalities. *Molecular Genetics & Genomic Medicine*, *6*, 294–300. | Target population- Not MZ Discordant Twins |
| Brunsdon, V.E., Colvert, E., Ames, C., Garnett, T., Gillan, N., Hallett, V., … & Happé, F. (2015). Exploring the cognitive features in children with autism spectrum disorder, their co-twins, and typically developing children within a population-based sample. *Journal of Child Psychology And Psychiatry*, *56*, 893–902. | Study design- Not a co-twin control design study |
| Castellheim, A., Lundström, S., Molin, M., Kuja-Halkola, R., Gillberg, C., & Gillberg, C. (2018). The role of general anesthesia on traits of neurodevelopmental disorders in a Swedish cohort of twins. *Journal of Child Psychology and Psychiatry*, *59*, 966–972. | Outcomes- No usable outcome measurement |
| Castelbaum, L., Sylvester, C. M., Zhang, Y., Yu, Q., & Constantino, J. N. (2020). On the Nature of Monozygotic Twin Concordance and Discordance for Autistic Trait Severity: A Quantitative Analysis. *Behavior Genetics, 50*(4), 263-272. | Study design- Not a co-twin control design study |
| de Zeeuw, E.L., van Beijsterveldt, C., Hoekstra, R.A., Bartels, M., & Boomsma, D.I. (2017). The etiology of autistic traits in preschoolers: a population-based twin study. *Journal of Child Psychology and Psychiatry*, *58*, 893–901. | Study design- Not a co-twin control design study |
| Deng, W., Zou, X., Deng, H., Li, J., Tang, C., Wang, X., & Guo, X. (2015). The Relationship Among Genetic Heritability, Environmental Effects, and Autism Spectrum Disorders: 37 Pairs of Ascertained Twin Study. *Journal of Child Neurology*, *30*, 1794–1799. | Study design- Not a co-twin control design study |
| Docherty, L.E., Rezwan, F.I., Poole, R.L., Turner, C.L., Kivuva, E., Maher, E.R., … & Mackay, D.J. (2015). Mutations in NLRP5 are associated with reproductive wastage and multilocus imprinting disorders in humans. *Nature Communications*, *6*, 8086. | Target population- MZ Twins did not have ASD diagnosis/traits |
| Dufek, J., Harry, J., Eggleston, J., & Hickman, R. (2018). Walking Mechanics and Movement Pattern Variability in Monozygotic Twins with Autism Spectrum Disorder. *Journal of Developmental and Physical Disabilities*, *30*, 793–805. | Target population- MZ twins concordant for clinical diagnosis of ASD |
| Dworzynski, K., Ronald, A., Hayiou‐Thomas, M.E., McEwan, F., Happé, F., Bolton, P.F., & Plomin, R. (2008). Developmental path between language and autistic‐like impairments: a twin study. *Infant and Child Development*, *17*, 121–136. | Study design- Not a co-twin control design study |
| Edelson, L.R., & Saudino, K.J. (2009). Genetic and environmental influences on autistic-like behaviors in 2-year-old twins. *Behavior Genetics*, *39*, 255–264. | Study design- Not a co-twin control design study |
| Gilmore, J. H., van Tol, J. J., Lewis Streicher, H., Williamson, K., Cohen, S. B., Greenwood, R. S., . . . Chescheir, N. C. (2001). Outcome in children with fetal mild ventriculomegaly: a case series. *Schizophrenia Research, 48*(2-3), 219-226. | Target population- Not diagnosed with DSM or ICD |
| Hallmayer, J., Cleveland, S., Torres, A., Phillips, J., Cohen, B., Torigoe, T., … & Risch, N. (2011). Genetic heritability and shared environmental factors among twin pairs with autism. *Archives of General Psychiatry*, *68*, 1095–1102 | Study design- Not a co-twin control design study |
| Hayretdag, C., Algedik, P., Ekmekci, C., Gunal, O., Agyuz, U., Yildirim, H., & Coskunpinar, E. (2020). Determination of genetic changes in etiology of autism spectrum disorder in twins by whole-exome sequencing. *Gene Reports*, *19*, 100618. | Target population- No MZ ASD discordant twin pairs |
| Hegarty, J. P., 2nd, Gu, M., Spielman, D. M., Cleveland, S. C., Hallmayer, J. F., Lazzeroni, L. C., . . . Hardan, A. Y. (2018). A proton MR spectroscopy study of the thalamus in twins with autism spectrum disorder. *Progress in Neuro-psychopharmacology & Biological Psychiatry, 81*, 153-160. | Target population- Not our target population |
| Hegarty II, J.P., Pegoraro, L., Lazzeroni, L.C., Raman, M.M., Hallmayer, J.F., Monterrey, J.C., … & Hardan, A.Y. (2019). Genetic and environmental influences on structural brain measures in twins with autism spectrum disorder. *Molecular Psychiatry*, 10.1038/s41380-018-0330-z. [Advance online publication] | Target population- Excluded MZ ASD discordant twin pairs from analyses |
| Hegarty, J.P., Lazzeroni, L.C., Raman, M.M., Pegoraro, L., Monterrey, J.C., Cleveland, S.C., … & Hardan, A.Y. (2020). Genetic and Environmental Influences on Lobar Brain Structures in Twins With Autism. *Cerebral Cortex*, *30*, 1946–1956. | Target population- Excluded MZ ASD discordant twin pairs from analyses |
| Hegarty II, J.P., Lazzeroni, L.C., Raman, M.M., Hallmayer, J.F., Cleveland, S.C., Wolke, O.N., … & Hardan, A. Y. (2020). Genetic and environmental influences on corticostriatal circuits in twins with autism. *Journal of Psychiatry & Neuroscience*, *45*, 188–197. | Target population- Excluded MZ ASD discordant twin pairs from analyses |
| Hu, V.W., Devlin, C.A., & Debski, J.J. (2019). ASD Phenotype-Genotype Associations in Concordant and Discordant Monozygotic and Dizygotic Twins Stratified by Severity of Autistic Traits. *International Journal of Molecular Sciences*, *20*, 3804. | Study design- Not a co-twin control design study |
| Ishijima, M., & Kurita, H. (2007). Brief report: identical male twins concordant for Asperger's disorder. *Journal of Autism and Developmental Disorders*, *37*, 386–389. | Target population- Not a MZ ASD discordant twin pair |
| Isaksson, J., Tammimies, K., Neufeld, J., Cauvet, É., Lundin, K., Buitelaar, J.K., … & EU-AIMS LEAP group (2018). EU-AIMS Longitudinal European Autism Project (LEAP): the autism twin cohort. *Molecular Autism*, *9*, 26. | Outcomes- No usable outcome data |
| Kates, W.R., Ikuta, I., & Burnette, C.P. (2009). Gyrification patterns in monozygotic twin pairs varying in discordance for autism. *Autism Research*, *2*, 267–278. | Target population- Compared MZ concordant and discordant pairs in analyses (no within pair analysis) |
| Kates, W.R., Burnette, C.P., Eliez, S., Strunge, L.A., Kaplan, D., Landa, R., … & Pearlson, G.D. (2004). Neuroanatomic variation in monozygotic twin pairs discordant for the narrow phenotype for autism. *The American Journal of Psychiatry*, *161*, 539–546. | Target population- Mixed concordant/discordant sample, but not within pair analysis |
| Kaur, K., Chauhan, V., Gu, F., & Chauhan, A. (2014). Bisphenol A induces oxidative stress and mitochondrial dysfunction in lymphoblasts from children with autism and unaffected siblings. *Free Radical Biology & Medicine*, *76*, 25–33. | Target population- Not MZ ASD discordant twin pairs |
| Kolevzon, A., Smith, C.J., Schmeidler, J., Buxbaum, J.D., & Silverman, J.M. (2004). Familial symptom domains in monozygotic siblings with autism. *American Journal of Medical Genetics. Part B, Neuropsychiatric Genetics*, *129B*, 76–81. | Target population- Not a MZ ASD discordant twin pair |
| Kramer, I., Lipkin, P.H., Marvin, A.R., & Law, P.A. (2015). A Genetic Multimutation Model of Autism Spectrum Disorder Fits Disparate Twin Concordance Data from the USA and Canada. *International Scholarly Research Notices*, *2015*, 519828. | Study design- Not a co-twin control design study |
| Le Couteur, A., Bailey, A., Goode, S., Pickles, A., Robertson, S., Gottesman, I., & Rutter, M. (1996). A broader phenotype of autism: the clinical spectrum in twins. *Journal of Child Psychology and Psychiatry*, *37*, 785–801. | Outcome- No usable outcome data |
| Losh, M., Esserman, D., Anckarsäter, H., Sullivan, P.F., & Lichtenstein, P. (2012). Lower birth weight indicates higher risk of autistic traits in discordant twin pairs. *Psychological Medicine*, *42*, 1091–1102. | Target population- Not diagnosed with DSM or ICD |
| Marrus, N., Glowinski, A.L., Jacob, T., Klin, A., Jones, W., Drain, C.E., … & Constantino, J.N. (2015). Rapid video-referenced ratings of reciprocal social behavior in toddlers: a twin study. *Journal of Child Psychology and Psychiatry*, *56*, 1338–1346. | Target population- Not MZ ASD discordant twin pairs |
| McKernan, E., Russo, N., Burnette, C., & Kates, W. (2017). ASD concordance of twins across DSM-IV-TR and DSM-5 diagnostic criteria. *Research in Autism Spectrum Disorders*, *41-42*, 51–56. | Study design- Not a co-twin control design study |
| Micalizzi, L., Ronald, A., & Saudino, K.J. (2016). A Genetically Informed Cross-Lagged Analysis of Autistic-Like Traits and Affective Problems in Early Childhood. *Journal of Abnormal Child Psychology*, *44*, 937–947. | Study design- Not a co-twin control design study |
| Mitchell, S.R., Reiss, A.L., Tatusko, D.H., Ikuta, I., Kazmerski, D.B., Botti, J.A., … & Kates, W.R. (2009). Neuroanatomic alterations and social and communication deficits in monozygotic twins discordant for autism disorder. *The American Journal of Psychiatry*, *166*, 917–925. | Target population- compared MZ concordant and discordant pairs in analyses (no within pair analysis) |
| Monterrey, J.C., Philips, J., Cleveland, S., Tanaka, S., Barnes, P., Hallmayer, J.F., … & Hardan, A.Y. (2017). Incidental brain MRI findings in an autism twin study. *Autism Research*, *10*, 113–120. | Study design- Not a co-twin control design study |
| Neuhaus, E., Kresse, A., Faja, S., Bernier, R.A., & Webb, S.J. (2016). Face processing among twins with and without autism: social correlates and twin concordance. *Social Cognitive and Affective Neuroscience*, *11*, 44–54. | Outcome- Did not indicate numbers of MZ from DZ Discordant pairs, or separate them by zygosity in analyses |
| Pua, E., Barton, S., Williams, K., Craig, J.M., & Seal, M.L. (2020). Individualised MRI training for paediatric neuroimaging: A child-focused approach. *Developmental Cognitive Neuroscience*, *41*, 100750. | Outcome- No usable outcome data |
| Rio, M., Royer, G., Gobin, S., de Blois, M.C., Ozilou, C., Bernheim, A., … & Malan, V. (2013). Monozygotic twins discordant for submicroscopic chromosomal anomalies in 2p25.3 region detected by array CGH. *Clinical Genetics*, *84*, 31–36. | Target population- Not diagnosed with DSM or ICD |
| Rosenberg, R.E., Law, J.K., Yenokyan, G., McGready, J., Kaufmann, W.E., & Law, P.A. (2009). Characteristics and concordance of autism spectrum disorders among 277 twin pairs. *Archives of Pediatrics & Adolescent Medicine*, *163*, 907–914. | Study design- Not a co-twin control design study |
| Rutherford M.D. (2005). A retrospective journal-based case study of an infant with autism and his twin. *Neurocase*, *11*, 129–137. | Target population-Cases were not monozygotic discordant twins |
| Trevarthen, C., & Daniel, S. (2005). Disorganized rhythm and synchrony: early signs of autism and Rett syndrome. *Brain & Development*, *27 Suppl 1*, S25–S34. | Target population- No validation of zygosity |
| Van't Westeinde, A., Cauvet, É., Toro, R., Kuja-Halkola, R., Neufeld, J., Mevel, K., & Bölte, S. (2019). Sex differences in brain structure: a twin study on restricted and repetitive behaviors in twin pairs with and without autism. *Molecular Autism*, *11*, 1. | Target population- Not our target population |
| Vukicevic, J., & Siegel, B. (1990). Pervasive developmental disorder in monozygotic twins. *Journal of the American Academy of Child and Adolescent Psychiatry*, *29*, 897–900. | Target population- MZ pervasive developmental disorder concordant twins |
| Yamasue, H., Ishijima, M., Abe, O., Sasaki, T., Yamada, H., Suga, M., . . . Kasai, K. (2005). Neuroanatomy in monozygotic twins with Asperger disorder discordant for comorbid depression. *Neurology, 65*(3), 491-492. | Target population- Not a MZ ASD discordant twin pair |

Note: Target population: not target population and/or no within pair analysis (i.e., studies of MZ discordant twins or case studies where both twins were diagnosed with ASD or had similar levels of clinical autism symptoms or autistic traits; studies solely comparing MZ discordant twins with either DZ discordant pairs and/or a control sample of typically developing twins and/or control individuals), not diagnosed with DSM or ICD (i.e., studies that failed to describe and/or use validated methods for the diagnosis of ASD or autistic traits/symptoms), or no validation of zygosity (i.e., studies that failed to describe validated methods of zygosity determination); Outcomes: no usable outcome data for phenotypic features; Study design: Not co-twin control design (e.g., studies focusing on heritability or concordance of diagnosis estimates alone).

**Table S2.** Data Extraction Elements.

| Data Extraction Elements |
| --- |
| Study author(s) and publication year |
| Name of dataset/cohort |
| Study temporality |
| Study setting (i.e., community or clinical) |
| Type of study (i.e., group analysis/cohort, case series, case study) |
| Zygosity and method to determine zygosity |
| Discordant for ASD or traits/symptoms and method for determination |
| Definition for dimensional discordance |
| Number of discordant pairs |
| Mean age/SD, age range |
| Female to male ratio |
| Socioeconomic status |
| Race/ethnicity |
| Nature of study |
| Variables of interest and method of measurement |
| Direction of statistical association and type of statistical analysis (if applicable) |
| Main results |
| Covariates included in statistical analyses |
| Newcastle-Ottawa Rating Scale (NOS) individual item scores and supporting justification |

**Figure S1.** Modified Newcastle-Ottawa Scale Ratings for All Included Articles.


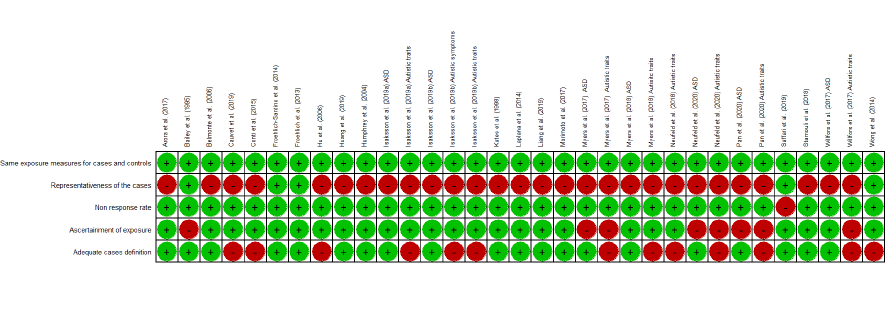


Ratings for all included articles using the modified, case-control version of the NOS. Ratings are based on selection of cases (i.e., adequate case definition and representativeness of cases) and exposure (i.e., ascertainment of exposure, same method of ascertainment, and non-response rate).

**Table S3.** Study Details (Country, Dataset, Temporality, and Setting).

| **Study** | **Country** | **Dataset (Name of Cohort)** | **Study temporality** | **Setting** |
| --- | --- | --- | --- | --- |
| Arora et al. (2017) | Sweden | RATSS | retrospective | community |
| Bailey et al. (1995) | United Kingdom | NR | retrospective | mixed clinical and community |
| Belmonte et al. (2006) | NR | NR | cross-sectional | community |
| Cauvet et al. (2019) | Sweden | RATSS | cross-sectional | community |
| Cauvet et al. (2020) | Sweden | RATSS | cross-sectional | community |
| Conti et al. (2015) | Italy | NR | cross-sectional | clinical |
| Froehlich et al. (2013) | California, United States | CATS | cross-sectional | community |
| Froehlich-Santino et al. (2014) | California, United States | CATS | retrospective | community |
| Hu et al. (2006) | United States | AGRE | cross-sectional | community |
| Huang et al. (2019) | China | CDBRC | cross-sectional | clinical |
| Humphrey et al. (2004) | Unknown | NR | longitudinal | clinical |
| Isaksson et al. (2019a) | Sweden | RATSS | cross-sectional | community |
| Isaksson et al. (2019b) | Sweden | RATSS | cross-sectional | community |
| Isaksson et al. (2019b) | Sweden | RATSS | cross-sectional | community |
| Kates et al. (1998) | United States | NR | cross-sectional | clinical |
| Laplana et al. (2014) | Spain | NR | cross-sectional | clinical |
| Liang et al. (2019) | China | NR | cross-sectional | clinical |
| Morimoto et al. (2017) | Japan | NR | cross-sectional | NR |
| Myers et al. (2017) | Sweden | RATSS | cross-sectional | community |
| Myers et al. (2018) | Sweden | RATSS | cross-sectional | community |
| Neufeld et al. (2018) | Sweden | RATSS | cross-sectional | community |
| Neufeld et al. (2020) | Sweden | RATSS | cross-sectional | community |
| Pan et al. (2020) | Sweden | RATSS | retrospective | community |
| Saffari et al. (2019) | United Kingdom | TEDS | cross-sectional | community |
| Stamouli et al. (2018 | Sweden | RATSS | cross-sectional | community |
| Willfors et al. (2017) | Sweden | RATSS | retrospective | community |
| Wong et al. (2014) | United Kingdom | TEDS | cross-sectional | community |

Note: RATSS- Roots of Autism and ADHD Twin Study in Sweden; NR- Not Reported, CATS- California Autism Twin Study; AGRE- Autism Genetic Resource Exchange; CDBRC- Child Development and Behavior Research Center; TEDS- Twins Early Development Study

**Table S4.** Study Methods (Zygosity, Diagnosis of ASD/Traits, Exposure/Outcome Measures and Methods).

| Study | Method to determine zygosity | Method to identify the diagnosis of ASD or traits/symptoms | Definition of dimensionally discordant | Exposure/Outcome Measures | Method to Identify Exposure/Outcome |
| --- | --- | --- | --- | --- | --- |
| Arora et al. (2017) | Genotyping | DSM-5, ADOS-2, ADI-R | NA | Fetal and postnatal Manganese, Zinc, and Lead level recorded in teeth | Laser ablation-inductively coupled plasma mass spectrometry (LA-ICP-MS) |
| Bailey et al. (1995) | Zygosity determined by typing nine blood groups | ICD-10, ADOS, ADI | NA | Biological hazards and differences | Standardized obstetric and perinatal histories taken from parents and collated with details from the birth records; if relevant, an epilepsy diagnostic interview administered |
| Belmonte et al. (2006) | NR | ADI-R and AQ | NA | Brain structure and function | sMRI and fMRI |
| Cauvet et al. (2019) | DNA testing or questionnaire | SRS-2 and ADOS Severity Score | NA | 1. Volumes of 96 regional gyri & cortical volume, surface area and thickness of 148 regions  2. Neuroanatomy (Cortical Volume, Cortical Thickness and Surface Area) | 1. Brain volume extraction & surface based analysis from T1-weighted structural images  2. Surface-based analysis |
| Cauvet et al. (2020) | DNA testing or questionnaire | DSM-5, ADOS-2, ADI-R | NA | Social Cognition | Movie for the Assessment of Social Cognition (MASC) |
| Conti et al. (2015) | Genomic sequencing | ADOS | NA | Structural connectivity between 66 cortical regions = 1122 tracts | MRI- high angular resolution diffusion imaging (HARDI) probabilistic tractography and extraction of fractional anisotropy per tract; counted tracts with more that 10% difference |
| Froehlich et al. (2013) | Genotyping | ADOS and ADI-R | NA | 1. Head circumference  2. Macrocephaly | 1. Direct measurement of head circumference with standard procedures (taken 3 times, max measurement recorded)  2. Defined as head circumference >97 % according to United States Head Circumference Growth Reference Charts |
| Froehlich-Santino et al. (2014) | Genotyping | ADOS and ADI-R | NA | Respiratory distress | Obtained from birth records through California Department of Public Health |
| Hegerty et al. (2018) | DNA: Nine short tandem repeat loci and the X/Y amelogenin were amplified and compared within twin pairs; concordance on all markers was considered MZ whereas discordance for at least one marker was considered DZ | ADOS and ADI-R |  | Neurometabolite ratios from the thalamus (main: N-acetyl aspartate, secondary: glutamate/glutamin; a marker of cellular membrane degradation/maturation and a glial cell marker) | MRI and MRS |
| Hu et al. (2006) | Genotyping | ADI-R (three co-twins with autistic traits diagnosed as "broad spectrum" or "not quite autistic" according to guidelines described by the Autism Genetic Resource Exchange (AGRE) repository) | NA | Gene expression | DNA microarray and quantitative RT-PCR of lymphoblastoid cell lines (LCL) derived from lymphocytes (from blood draw) |
| Huang et al. (2019) | Genotyping | DSM-5 and ADOS | NA | Genetic variation: SNVs, small insertions or deletions (indels), and copy number variations (CNVs). | Whole genome sequencing (from blood)-HaplotypeCaller (HC) used to detect SNVs and small indels (< 65 nt). GATK Variant Quality Score Recalibration (VQSR) used to filter spurious SNVs and indels due to sequencing errors and mapping artifacts. CNV analysis performed with DELLY. |
| Humphrey et al. (2004) | DNA testing: Nine highly polymorphic DNA markers were identical in both twins | ADOS, ADI-R, ICD-10, DSM-IV | NA | 1. Cognitive function  2. Tuber location/number  3. Epilepsy history | 1. Mullen Scales of Early Learning (MSEL)  2. MRI  3. Parent report and medical history |
| Isaksson et al. (2019a) | DNA testing | DSM-5, ADOS-2, ADI-R, SRS-2 | Not defined for SRS-2 | Social Cognition ability | Reading the Mind in the Eyes Test (RMET) |
| Isaksson et al. (2019b) | Panel of 48 single nucleotide polymorphisms | DSM-5, ADOS-2, ADI-R, SRS | Not defined for SRS-2 or ADOS | Social cognition | Movie for the Assessment of Social Cognition (MASC) and MASC subscales |
| Kates et al. (1998) | DNA fingerprinting probes | ADOS and ADI-R | NA | Total and regional brain volumes plus CSF volume | MRI: The total brain, frontal lobe, temporal lobe, superior temporal gyrus, amygdala, and hippocampus measured manually on coronal image stacks |
| Laplana et al. (2014) | Genotyping | ADI-R (both twins) and ADOS (only on less affected twin), DSM-4 | NA | Germinal and somatic CNV regions | Array comparative genome hybridization (CGH) performed in three different tissues: blood, saliva, and hair follicle |
| Liang et al. (2019) | Genotyping | DSM-5, ADOS | NA | DNA methylation | Infinium 450K array (3 pairs) and RRBS (2 pairs). Pyrosequencing employed to further validate differences |
| Morimoto et al. (2017) | Genotyping | ICD-10, DSM-5 | NA | Discordant DNA variants (SNVs, insertion/deletion (Indels), and CNVs) | Genome Analysis Toolkit used to perform local realignment (Genome Analysis Toolkit IndelRealigner) and variant call (Genome Analysis Toolkit HaplotypeCaller) implemented an in-house workflow management tool. Findings validated by capillary sequencing and deep sequencing using MiSeq. WES data applied to eXome-Hidden Markov Model10 analysis for detecting discordant CNVs, validated by Droplet Digital PCR (ddPCR). |
| Myers et al. (2017) | Genotyping | DSM-5, ADOS-2, ADI-R, SRS-2 | Not defined for SRS-2 | Number and Type of Minor Physical Anomalies (MPAs) | Two independent raters with study developed checklist based on standardized dysmorphology references |
| Myers et al. (2018) | Blood/saliva for genotyping genotyping with Infinium Human-CoreExome chip (Illumina) or using a panel of 47 validated single nucleotide polymorphisms | DSM-5, ADOS-2, ADI-R,  SRS-2 | Not defined for SRS-2 | overall hand 2D:4D ratio | Two independent raters using digital measurement system (ImageJ) |
| Neufeld et al. (2018) | NR | SRS-2 | Not defined for SRS-2 | Resting state connectivity in default mode network (DMN) and salience network (SN) | MRI |
| Neufeld et al. (2020) | Panel of 48 single nucleotide polymorphisms for 128 pairs or 4-item zygosity questionnaire | DSM-5, ADOS-2, ADI-R, SRS-2 | 1 point difference on SRS | 1. Global processing  2. Local processing | 1. Fragmented Pictures Test (FPT)  2. Embedded Figures Test (EFT) and the Block Design Test (BDT) |
| Pan et al. (2020) | DNA testing with saliva or whole-blood sample | DSM-5, ADOS-2, ADI-R, SRS-2 | intra-pair difference on the total score of the Social Responsiveness Scale-2 (SRS-2) of at least 6 points, corresponding to 1 standard error of measurement | Neurological problems | Medical History Questionnaire |
| Saffari et al. (2019) | Genotyping | ADOS, ADI-R, CASR | NA | Gene expression (transcription differences) | RNA sequencing on whole blood samples (feature counts program from the subread package v1.4.6 used to summarize and quantify the mapped reads) |
| Stamouli et al. (2018 | Genotyping | DSM-5, ADOS-2, ADI-R | NA | Rare CNVs | Illumina Infinium PsychArray and derived through 4 algorithims: PennCNV, QuantiSNP, iPattern, and iPsychCNV |
| Willfors et al. (2017) | Genotyping of saliva or whole-blood | DSM-5, ADOS-2, ADI-R, SRS-2 | At least 1 point intra-pair difference on SRS-2 total score | Differences in early medical events | Parent reported questionnaire on early medical history with multisource medical records to identify medical history events to generate cumulative load of early medical events for each individual (based on 31 factors) |
| Wong et al. (2014) | Genotyping | CAST | At least 1 point intra-pair difference on subscale scores of CAST | DNA methylation differences | Genome-wide analysis of DNA methylation |

Notes: DSM-5- Diagnostic and Statistical Manual of Mental Disorders, 5^th^ edition; DSM-4- Diagnostic and Statistical Manual of Mental Disorders 4^th^ edition; ADOS- Autism Diagnostic Observation Schedule; ADOS-2- Autism Diagnostic Observation Schedule-2; ADI- Autism Diagnostic Interview; ADI-R- Autism Diagnostic Interview-Revised; ICD-10- International Classification of Diseases-10; AQ- Autism Quotient; AGRE- Autism Genetic Resource Exchange; SRS-2- Social Responsiveness Scale-2; CAST- Childhood Autism Spectrum Test; MRI- Magnetic Resonance Imaging; sMRI- Structural Magnetic Resonance Imaging; fMRI- Functional Magnetic Resonance Imaging; MRS- Magnetic Resonance Spectroscopy; DNA- Deoxyribonucleic Acid; RNA- Ribonucleic Acid; CNV- Copy Number Variant; WES- Whole Exome Sequencing; PCR-Polymerase Chain Reaction; RRBS- Reduced Representation Bisulfite Sequencing; NA: Not Applicable; NR= Not Reported,
